# News and Coming Events

**Published:** 1907-02-23

**Authors:** 


### NEWS AND COMING EVENTS.

MAJOR P. B. WALKER, J.P., has been elected President of the Dewsbury General Infirmary.

AT a public meeting held at Maesteg last week it was decided to form a Committee to consider the question of establishing a cottage hospital in the town.

MR. A. BOAKE, a trustee of the Children's Sanatorium for Consumption, at Holt, Norfolk, has promised a sum of £500 for endowment of the institution.

AT the annual meeting of the St. Mark's Hospital for Fistula Sir Richard Martin, Bart., the Treasurer, stated that the sum of £1,000 in new annual subscriptions is essential for the maintenance of the hospital.

AT the meeting of the Incorporated Society of Medical Officers of Health to be held on March 8, Dr. O'Connor will read a paper on "Combined Sanitary Districts." The meetings of the Society are held at No. 1 Upper Montague Street, W.C.

AT the annual meeting of the Reading Dispensary the President, Mr. May, suggested that an infant department, similar to that existing at Ghent in Belgium, should be established in connection with the dispensary. The proposal is having the attention of the Committee.

THE Hull Corporation has decided to build a new city abattoir. The building will have lairage for 200 beasts, 600 sheep, and 200 pigs, with ample provision for further extension. The principal slaughter hall will be 91 feet by 36 feet, with four bays, each having two killing rings. The estimated cost of the abattoir is £33,000.

SIR WILLIAM BROADBENT, one of the founders of the "Société de l'Entente Cordiale Médicale," has been obliged, owing to ill-health, to cancel his arrangements for a course of medical lectures in Paris. The association has arranged to open its series of lectures in May next with a course of clinical demonstrations on "Aphasia" by Dr. Pierre Marie.

THE sixteenth edition of the "Local Government Annual and Official Directory," which has just been published, is, as usual, a mine of information on the details of the various metropolitan and suburban boards, councils, committees, corporations, and combined authorities. In addition, it gives a useful calendar in the shape of "The Local Government Remembrancer."

WE called attention in our issue of February 9, under the title "Strength in Little Things," to the wonderful character and results of an appeal issued by a lady in Worcestershire. After receiving some twelve copies of the appeal literature in question by various posts in stamped envelopes we have now received No. 13, which is unstamped, for which double postage was inadvertently paid on delivery. We hope the supply has now come to an end so far as we are concerned.

AN interesting series of experiments has been carried out at the Plague Research Laboratory at Bombay with a view to testing the comparative germicidal properties of pure nickel and nickel alloy, and to determine whether plague may possibly be conveyed by coins. The results show that all the coins had considerable bactericidal action on the plague bacillus, for they rendered sterile a highly virulent strain of that germ within twenty-four hours.

THE Royal Commission on Vivisection has issued its first report, consisting of the evidence so far taken.

THE fund started by Sir George White in aid of the Bristol Royal Infirmary now stands at £50,000.

AN International Exhibition of Professional Newspapers and Periodicals will be held in May at Copenhagen.

LORD ROTHSCHILD has accepted the office of President of the Royal Buckinghamshire Hospital for another year.

THE International Congress of Laryngo-Rhinology will be held in Vienna on April 21, 1908. Professor Chiari will preside.

LORD KINNOULL has offered the directors of the Perth Infirmary a site for the proposed new buildings at a nominal feu duty.

AN exhibition of urban cottages and homesteads for small holdings is to be held at the Garden City, Letchworth, this summer.

THE International Conference of the Red Cross Society will be held in London in May next, the exact date not having been definitely fixed as yet.

The annual meeting of the governors and subscribers of Queen Charlotte's Lying-in Hospital, Marylebone Road, N.W., will take place on Monday, February 25, at 3 P.M. at the hospital.

MR. JAMES TAYLOR, who has been actively connected with the Chester Infirmary for the last half-century, has resigned his post as senior surgeon, and has been appointed honorary consulting surgeon to the institution.

THE annual Court of Governors of the Royal Free Hospital, Gray's Inn Road, W.C., will be held at the hospital on Monday next, February 25. The Right Hon. the Earl of Sandwich will take the chair at 4.15 o'clock.

THE annual general meeting of the Association of Certificated Dispensers will be held in the Apothecaries' Hall on Thursday, February 28, at 7.30 P.M. Address by F. S. Toogood, Esq., M.D.(Lond.), barrister-at-law.

DURING the past year 120 cases were treated in the Samuel Thompson Ward (Tropical Ward) of the Royal Southern Hospital, Liverpool. Among these were cases of malaria, beri-beri, dysentery, leprosy, and sprue, and the patients included members of sixteen different nationalities.

A CORRESPONDENT in the *Rugby News* suggests the starting of an "In Memoriam" fund in connection with the local hospital—"a fund wholly reserved for sums not exceeding what one would spend in purchasing a wreath in memory of a departed friend."

THE German Congress of Internal Medicine, under the presidency of Professor Von Leyden, meets this year at Wiesbaden on April 15. The Congress, which usually lasts for a few days only, is attended by all the prominent physicians and clinicians, and the programme of papers and discussions is generally a very interesting one. This year such subjects as New Methods of Clinical Diagnosis, Chronic Permanganate Poisoning, and the Ætiology and Treatment of Migraine, figure on the Agenda Paper.

